# Novel Antifungals and *Aspergillus* Section *Terrei* with Potpourri Susceptibility Profiles to Conventional Antifungals

**DOI:** 10.3390/jof9060649

**Published:** 2023-06-06

**Authors:** Roya Vahedi-Shahandashti, Jos Houbraken, Mike Birch, Cornelia Lass-Flörl

**Affiliations:** 1 Institute of Hygiene and Medical Microbiology, Medical University of Innsbruck, 6020 Innsbruck, Austria; roya.vahedi@i-med.ac.at; 2 Westerdijk Fungal Biodiversity Institute, 3584 CT Utrecht, The Netherlands; j.houbraken@wi.knaw.nl; 3 F2G Ltd., Manchester M30 0LX, UK; mbirch@f2g.com

**Keywords:** new antifungals, *Aspergillus terreus*, aspergillosis, antifungal susceptibility test, EUCAST, ibrexafungerp, manogepix, olorofim, rezafungin, resistance

## Abstract

The epidemiology of invasive fungal infections (IFIs) is currently changing, driven by aggressive immunosuppressive therapy, leading to an expanded spectrum of patients at risk of IFIs. Aspergillosis is a leading cause of IFIs, which usually affects immunocompromised patients. There are a limited number of antifungal medications available for treating IFIs, and their effectiveness is often hindered by rising resistance rates and practical limitations. Consequently, new antifungals, especially those with novel mechanisms of action, are increasingly required. This study assessed the activity of four novel antifungal agents with different mechanisms of activity, namely, manogepix, rezafungin, ibrexafungerp, and olorofim, against 100 isolates of *Aspergillus* section *Terrei*, containing amphotericin-B (AmB)-wildtype/non-wildtype and azole-susceptible/-resistant strains, according to the European Committee on Antimicrobial Susceptibility Testing (EUCAST) method. In general, all tested agents showed potent and consistent activity against the tested isolates, exhibiting geometric mean (GM) and minimum effective concentration (MEC)/minimum inhibitory concentration (MIC) ranges, respectively, as follows: manogepix (0.048 mg/L, 0.032–0.5 mg/L), rezafungin (0.020 mg/L, 0.016–0.5 mg/L), ibrexafungerp (0.071 mg/L, 0.032–2 mg/L), and olorofim (0.008 mg/L, 0.008–0.032 mg/L). In terms of MIC90/MEC90, olorofim had the lowest values (0.008 mg/L), followed by rezafungin (0.032 mg/L), manogepix (0.125 mg/L), and ibrexafungerp (0.25 mg/L). All the antifungals tested demonstrated promising in vitro activity against *Aspergillus* section *Terrei*, including *A. terreus* as well as azole-resistant and AmB-non-wildtype cryptic species.

## 1. Introduction

There is a growing trend of fungal infections affecting immuno-compromised and medically compromised patients [1,2]. The treatment of invasive fungal infections (IFIs), including invasive aspergillosis (IA), has remained challenging due to several factors, specifically the limitations of the currently available antifungal therapies and changing epidemiology [3,4]. *A. terreus* is the third or fourth most common etiological agent of IA, depending on the geographical region [5]. This species has a unique clinical position among the opportunistic pathogenic *Aspergillus* species due to its relatively high mortality rate and reduced susceptibility to amphotericin B (AmB), making treatment challenging [6,7,8,9]. Currently, voriconazole remains the first therapeutic choice for aspergillosis, followed by other substituted agents, such as isavuconazole (ISA), liposomal AmB (L-AmB), and voriconazole (VRC) with an echinocandin [10]. In addition to the limited therapeutic options available, azole-resistant *A. terreus* and related species, along with the tolerance phenomenon, threaten the current pipeline of antifungals [11,12,13,14].

New generations of antifungals are needed to combat the rapidly rising levels of resistance and their associated clinical failures [15]. The development of antifungal drugs has stagnated in the past two decades, with only ISA having been introduced [16]. Although ISA has a broader spectrum than VRC and fewer drug-related side effects, it still displays cross-resistance with other azoles [17]. Even though antifungal drug development is a lengthy process, it addresses the consequences of limited drug classes. Several antifungals are currently being developed in clinical trials and have received substantial support from pharmaceutical companies [18].

In the present study, the in vitro activity of some promising new drugs in development was analyzed, including ibrexafungerp, manogepix, olorofim, and rezafungin. Manogepix (formerly E1210) is the active component of fosmanogepix, a novel first-in-class broad-spectrum antifungal agent that inhibits the activity of the Gwt1 enzyme, which is involved in the biosynthesis of glycosylphosphatidylinositol(GPI) anchors, an essential component of the fungal cell wall [19,20]. This leads to defects in various steps of cell wall biosynthesis with the accompanying inhibition of cell wall growth, hyphal elongation, and the attachment of fungal cells to biological substrates [20]. Manogepix has been shown to have broad-spectrum activity against various molds and yeasts [19]. Ibrexafungerp (formerly SCY-078), a semisynthetic derivative of enfumafungin, is a potent inhibitor of fungal β-(1,3)-D-glucan synthases [21], with promising activity against *Aspergillus* and *Candida* species. Olorofim (formerly F901318), a new antifungal agent with a novel selective activity, inhibits fungal dihydroorotate dehydrogenase(DHODH), thus halting de novo pyrimidine biosynthesis and, ultimately, DNA synthesis, cell growth, and division [22,23]. The cyclic hexapeptide rezafungin (formerly CD101), which is structurally similar to anidulafungin, is an echinocandin that is highly active against *Aspergillus* [22]. The current study aimed to evaluate the in vitro activity of the above-mentioned new antifungals against a collection of *Aspergillus* section *Terrei* isolates, including AmB-wildtype/non-wildtype and azole-susceptible/-resistant *A. terreus* sensu stricto (s.s.) and related species, using the European Committee on Antimicrobial Susceptibility Testing (EUCAST) reference method.

## 2. Materials and Methods

**Figure 1:**
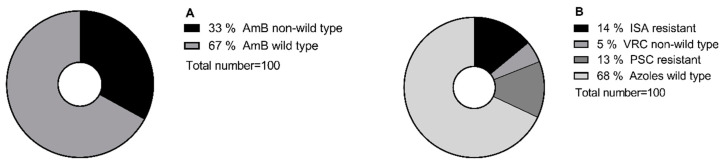
Pie chart illustrating the percentage of (**A**) AmB-wildtype/non-wildtype; (**B**) ISA- and PSC-resistant/-susceptible; and VRC-wildtype/non-wildtype isolates, according to the clinical breakpoint and Epidemiological cutoff values defined by EUCAST (https://www.eucast.org/mic_and_zone_distributions_and_ecoffs, (accessed on 18 January 2022); https://www.eucast.org/astoffungi/clinicalbreakpointsforantifungals, (accessed on 18 January 2022). PSC; posaconazole, VRC; voriconazole, ISA; isavuconazole, AmB; amphotericin B.

A total of 100 molecular-identified *Aspergillus* section *Terrei* isolates, including *A. terreus* s.s. (*n* = 30), *A. citrinoterreus* (*n* = 9), *A. alabamensis* (*n* = 7), *A. hortae* (syn. *A. hortai*; *n* = 6), *A. carneus* (*n* = 6), *A. niveus* (*n* = 6), *A. aureoterreus* (*n* = 5), *A. neoindicus* (*n* = 5), *A. iranicus* (*n* = 5), *A. neoafricanus* (*n* = 4), *A. pseudoterreus* (*n* = 4), *A. allahabadi* (*n* = 4), *A. floccosus* (*n* = 2), *A. barbosae* (*n* = 2), *A. bicephalus* (*n* = 1), *A. ambiguus* (*n* = 1), and *A. microcysticus* (*n* = 1), were analyzed. The isolate collection included strains that were previously obtained and included in the ISHAM-ECMM-EFISG TerrNet Study (www.isham.org/working-groups/aspergillus-terreus, (accessed on 24 February 2017)) [24] and those preserved in the CBS biobank housed at the Westerdijk Fungal Biodiversity Institute, Utrecht, the Netherlands. Strains were identified as previously described [13,25]. A selection of non-wildtype/wildtype and resistant/susceptible isolates was conducted based on the susceptibility profiles of the tested conventional antifungals (AmB, ISA, VRC, posaconazole (PSC)) (Figure 1). In total, 10% of selected isolates showed cross-resistance to the tested conventional antifungals.

Isolates from 10% glycerol frozen stocks (−80 °C) were cultured on malt extract agar (Carl Roth, Karlsruhe, Germany) at 37 °C for up to 5 days, and the spores were harvested by applying spore suspension buffer (0.9% NaCl, 0.01% Tween 20 (Sigma-P1379)). Antifungal susceptibility testing was performed according to the broth microdilution method of EUCAST [26]. The antifungals used were ibrexafungerp (range 0.03–16 mg/L; Scynexis, Inc., Jersey City, NJ, USA), olorofim (range 0.008–4 mg/L; F2G Ltd., Manchester, UK), rezafungin (range 0.01–8 mg/L; MedChemExpress, Sollentuna, Sweden), and manogepix (range 0.03–16 mg/L; MedChemExpress, Sollentuna, Sweden). The minimum inhibitory concentration (MIC), the concentration at which no hyphal growth was detected, was assessed for olorofim, and for the rest of the tested agents, the minimal effective concentration (MEC), which markedly altered hyphal growth with blunted colonies, was assessed. A final reading of the MIC results was performed with a stereoscope after 48 h. The geometric mean (GM), MIC_50_/MEC_50_ (MIC/MEC causing inhibition of 50% of the isolates tested), and MIC_90_/MEC_90_ (MIC/MEC causing inhibition of 90% of the isolates tested) were calculated.

## 3. Results

**Figure 2:**
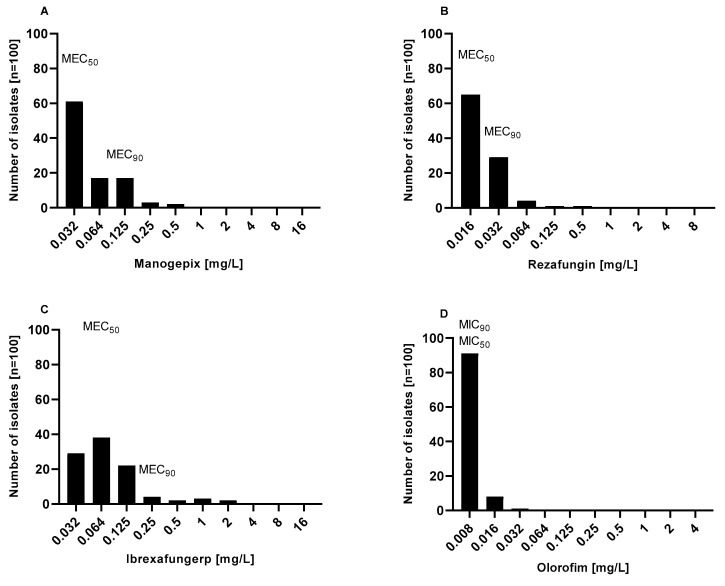
Distribution of minimum inhibitory concentrations (MICs) and minimum effective concentrations (MECs) of (**A**) manogepix, (**B**) rezafungin, (**C**) ibrexafungerp, and (**D**) olorofim against *Aspergillus* section *Terrei* (*n* = 100).

**Figure 3:**
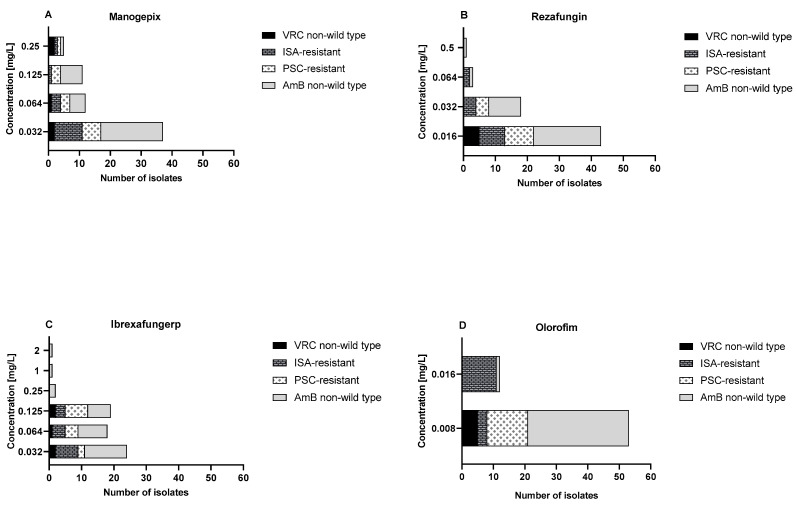
The activity of the tested antifungals, (**A**) manogepix, (**B**) rezafungin, (**C**) ibrexafungerp, and (**D**) olorofim, with a focus on AmB-non-wildtype (*n* = 33), ISA-resistant (*n* = 14), PSC-resistant (*n* = 13), and VRC-non-wildtype (*n* = 5) isolates of *Aspergillus* section *Terrei*. PSC; posaconazole, VRC; voriconazole, ISA; isavuconazole, AmB; amphotericin B.

The MIC distribution and in vitro susceptibility testing results of manogepix, rezafungin, ibrexafungerp, and olorofim against the 100 *Aspergillus* section *Terrei* isolates, including those with reduced susceptibility to AmB and resistance to azoles, are shown in Figure 2 and Figure 3 and Table 1.

Manogepix demonstrated potent in vitro activity against all tested isolates, as shown in Figure 2A, with MECs ranging from 0.032 to 0.5 mg/L, and the MEC_50_ and MEC_90_ values of 0.032 and 0.125 mg/L, respectively. Considering the species separately (Table 1), *A. citrinoterreus* and *A. bicephalus* demonstrated the highest MECs range (0.032–0.5 and 0.5 mg/L, respectively), and *A. carneus* and *A. niveus* the highest GM (both 0.086 mg/L). Furthermore, manogepix displayed potential activity at the lowest concentration (0.032 mg/L) against the majority of resistant/non-wildtype isolates (Figure 3A). The MEC range, MEC_50_, and MEC_90_ values of rezafungin were 0.016 to 0.5 mg/L, 0.016 mg/L, and 0.5 mg/L, respectively, against all tested *Aspergillus* (Figure 2B). Among all tested species, *A. carneus* showed the highest MEC range and GM for rezafungin (0.016–0.5 and 0.026 mg/L, respectively) (Table 1). Rezafungin inhibited most isolates at the lowest concentration, 0.016 mg/L, when focusing on resistant/non-wildtype isolates (Figure 3B). Ibrexafungerp yielded MEC range, MEC_50_, and MEC_90_ values of 0.03 to 2 mg/L, 0.06 mg/L, and 0.25 mg/L, respectively (Figure 2C). As compared to all other tested species, *A. citrinoterreus*, and *A. terreus* s.s, the most clinically isolated species, displayed the highest MEC range (both 0.032–2 mg/L), and *A. allahabadi* showed the highest GM (0.087 mg/L) (Table 1). According to the results, ibrexafungerp exhibited promising inhibitory activity at the lowest concentration range tested (0.032–0.06 mg/L) against most of the non-wildtype and resistant isolates (Figure 3C). Olorofim showed a high activity against all tested *Aspergillus* section *Terrei* isolates, exhibiting an MIC range, MEC_50_, and MEC_90_ values of 0.008–0.032 mg/L, 0.008 mg/L, and 0.008 mg/L, respectively (Figure 2D). Comparatively, *A. neoindicus* had the highest MIC range for olorofim (0.008–0.032 mg/L), and *A. iranicus* showed the highest GM (0.012 mg/L) (Table 1). Considering non-wildtype/resistant isolates separately, olorofim showed a significant inhibitory effect at the lowest concentration tested (0.008–0.016 mg/L) (Figure 3D).

**Table 1:** MIC values, ranges, and GMs for olorofim and MEC values, ranges, and GMs for ibrexafungerp, manogepix, and rezafungin against azole-susceptible/-resistant and AmB-wildtype/non-wildtype *Aspergillus* section *Terrei* (*n* = 100), as determined via the EUCAST broth microdilution method. MIC50/MEC50 and MIC90/MEC90 stand for MICs/MECs inhibiting ≥50% and ≥90% of the strains, respectively. The GM (geometric mean) is shown for species with at least four isolates or more.

*Aspergillus* Section *Terrei* (no.)	MEC Range (mg/L)/(MEC GM)			MIC Range (mg/L)/(MIC GM)
	Manogepix	Rezafungin	Ibrexafungerp	Olorofim
*A. alabamensis* (*n* = 7)	0.032/0.03	0.016–0.032/0.018	0.03–0.05/0.074	0.008/0.008
*A. allahabadii* (*n* = 4)	0.032/0.03	0.016–0.032/0.017	0.06–0.125/0.087	0.008–0.016/0.009
*A. ambiguus* (*n* = 1)	0.032/-	0.016/-	0.06/-	0.008/-
*A. aureoterreus* (*n* = 5)	0.032–0.125/0.045	0.016–0.032/0.019	0.03–0.125/0.053	0.008/0.008
*A. barbosae* (*n* = 2)	0.032/-	0.016/-	0.06–0.125/-	0.008/-
*A. bicephalus* (*n* = 1)	0.5/-	0.016/-	0.03/-	0.008/-
*A. carneus* (*n* = 6)	0.032–0.25/0.086	0.016–0.5/0.026	0.03–0.25/0.061	0.008–0.016/0.011
*A. citrinoterreus* (*n* = 9)	0.032–0.5/0.070	0.016–0.032/0.018	0.03–2/0.076	0.008/0.008
*A. floccosus* (*n* = 2)	0.064–0.125/-	0.016–0.032/-	0.03–0.25/-	0.008–0.016/-
*A. hortai* (*n* = 6)	0.032–0.125/0.038	0.016–0.125/0.023	0.06–1/0.155	0.008/0.008
*A. iranicus* (*n* = 5)	0.032–0.06/0.039	0.016–0.032/0.019	0.03–0.06/0.045	0.008–0.016/0.012
*A. micocysticus* (*n* = 1)	0.032/-	0.016/-	0.03/-	0.008/-
*A. neoafricanus* (*n* = 5)	0.032–0.125/0.039	0.016–0.06/0.025	0.03–1/0.173	0.008/0.008
*A. neoindicus* (*n* = 5)	0.032–0.125/0.045	0.016–0.032/0.023	0.03–0.125/0.06	0.008–0.032/0.01
*A. niveus (n* = 6)	0.032–0.25/0.086	0.016–0.06/0.023	0.3–0.125/0.061	0.008/0.008
*A. pseudoterreus* (*n* = 4)	0.032–0.06/0.035	0.016–0.032/0.017	0.06/0.06	0.008/0.008
*A. recifensis* (*n* = 2)	0.032–0.125/-	0.032/-	0.125/-	0.008/-
*A. terreus s.s* (*n* = 30)	0.032–0.125/0.044	0.016–0.06/0.019	0.03–2/0.067	0.008/0.008
*All isolates* (*n* = 100)				
GM	0.048	0.020	0.071	0.008
Range	0.032–0.5	0.016–0.5	0.032–2	0.008–0.032
MEC 50/90	0.032/0.125	0.016/0.032	0.064/0.25	-
MIC50/90	-	-	-	0.008/0.008

Overall, all agents demonstrated promising activity against tested isolates and considering GM of all species together, the lowest value was assigned to olorofim, followed by rezafungin, manogepix, and ibrexafungerp (0.008 mg/L, 0.020 mg/L, 0.048 mg/L, and 0.071 mg/L, respectively).

## 4. Discussion

The mortality rate for aspergillosis infections remains high, despite improved diagnosis and prophylaxis [27]. There are currently four major classes of antifungal agents used to treat systemic mycoses: polyenes, azoles, echinocandins, and flucytosine [28]. The effectiveness of the present antifungals is affected by their toxicity, drug–drug interactions, variable pharmacokinetics, and reduced bioavailability [28]. The emergence of drug resistance has introduced further limitations [29]. For IA, VRC is the first line of treatment; alternatives include ISA, L-AmB, and VRC with an echinocandin [30]. Resistance to azoles, the first-line treatment, has grown at an alarming rate in the last decade, posing a serious challenge to the effective management of aspergillosis [29,31]. The identification of antifungal resistance relies on susceptibility testing, identifying MICs to define susceptibility or resistance. Several factors further complicate treatment and lead to poor outcomes, such as method dependency of the susceptibility testing results and, consequently, discrepancies between in vitro and in vivo outcomes, as well as tolerance and persistence phenomena, which are not detectable using reference susceptibility testing methods [14,32,33]. Therefore, the reduction in the currently limited antifungal arsenal has led to patient management complications and higher mortality due to resistant isolates, which call for new antifungal agents and therapeutic approaches [3]. Since *A. terreus* is naturally less susceptible to AmB, azole resistance in this species is of particular concern, as this could lead to a loss of two primary lines of treatment [7,13]. Furthermore, some less common species of section *Terrei* exhibit high azole MICs, which, if not identified before antifungal therapy, may cause clinical failure [32]. Thus, in this study, novel antifungals were tested against nearly all currently accepted species of section *Terrei*, including isolates with reduced susceptibility to conventional antifungals.

Similar to previous studies [34,35], manogepix exhibited encouraging activity against all the tested *Aspergillus* spp. isolates, including AmB-non-wildtype and azole-resistant isolates. Manogepix inhibited all the tested isolates at 0.5 mg/L (MEC_50_, 0.032 mg/L; MEC_90_, 0.125 mg/L) (Figure 2A and Figure 3A, and Table 1). Despite the similar MEC_50_ and MEC_90_ values of *A. terreus* s.s. and *A. terreus* non-s.s., when compared separately, all *A. terreus* s.s. were inhibited at 0.125 mg/L, while all *A. terreus* non-s.s. were suppressed at 0.5 mg/L. As observed in our study, a study of clinical isolates from Spanish patients found manogepix to be effective against cryptic *Aspergillus* species, including those resistant to PSC and AmB [36]. Furthermore, according to a recent study, the in vivo combination of manogepix and L-AmB showed synergistic effects in reducing the invasive pulmonary aspergillosis fungal burden and improving survival [37]. Synergistic effects with L-AmB may have greater utility in cases where azole resistance is suspected.

Rezafungin demonstrated significant in vitro activity against all the tested isolates at 0.5 mg/L (MEC_50_, 0.016 mg/L; MEC_90_, 0.032 mg/L) (Figure 2B and Figure 3B, and Table 1). The rezafungin MECs were higher for *A. terreus* non-s.s. than *A. terreus* s.s., with 0.5 mg/L (MEC_50_, 0.016 mg/L; MEC_90_, 0.032 mg/L) and 0.06 mg/L (MEC_50_, 0.016 mg/L; MEC_90_, 0.032 mg/L), respectively. The prolonged half-life of rezafungin in vivo [38], along with its potent in vitro activity against *Aspergillus* spp. [39], suggests that it may be beneficial in treating patients with infections caused by azole-resistant *Aspergillus*. However, it should be noted that monotherapy with an echinocandin is not currently recommended as a primary treatment for IA. To determine whether this potent in vitro activity would accelerate with combination therapy and whether it would translate into in vivo efficacy against infections caused by resistant *Aspergillus* isolates, additional studies are warranted.

Ibrexafungerp, the new beta-glucan synthase inhibitor, showed promising antifungal activity in vitro against the tested *Aspergillus* section *Terrei*, with an MEC of 2 mg/L (MEC_50_, 0.06 mg/L; MEC_90_, 0.25 mg/L) (Figure 2C and Figure 3C, and Table 1). There were no significant differences between the MECs of *A. terreus* s.s., at 2 mg/L (MEC_50_, 0.064 mg/L; MEC_90_, 0.125 mg/L), and *A. terreus* non-s.s., at 2 mg/L (MEC_50_, 0.064 mg/L; MEC_90_, 0.25 mg/L). Ibrexafungerp was previously shown to have in vitro and in vivo activity against *Aspergillus* species, including azole-resistant and caspofungin-resistant strains, a finding which is consistent with this study (Figure 2 and Figure 3) [40,41]. Furthermore, the synergistic effect of ibrexafungerp in combination with ISA, VRC, and AmB was demonstrated [42]. These results are likely to increase the appeal of using ibrexafungerp in combination with other agents for infections that are difficult to treat.

The strong activity of olorofim against the tested *Aspergillus* section *Terrei* was confirmed, including those species that showed reduced susceptibility to AmB and/or azoles (Figure 2D and Figure 3D, and Table 1). Olorofim had the lowest MICs at 0.032 mg/L (MEC50 and MEC90, both at 0.008 mg/L), with no differences between *A. terreus* s.s. and *A. terreus* non-s.s. In addition to the present study, other studies have also shown that olorofim is effective against azole-resistant *A. fumigatus* in vitro and in vivo in murine models of invasive pulmonary aspergillosis [22]. Additionally, this new drug has shown activity against other common *Aspergillus* species, including *A. terreus* [43,44,45]. Olorofim’s activity was retained against isolates showing resistance to azoles and/or AmB, and given its entirely different targeting of the azoles, cross-resistance would not be expected.

In conclusion, a set of novel antifungals (manogepix, rezafungin, ibrexafungerp, and olorofim) were demonstrated to have promising and consistent in vitro activity against nearly all currently accepted species of *Aspergillus* section *Terrei*, regardless of azole and AmB resistance. The development of novel agents could play a pivotal role in treating multi-resistant mold infections, including azole-resistant aspergillosis.

## Data Availability

All data are provided in this manuscript.
